# Living in a Hostile World: Inflammation, New Drug Development, and Coronavirus

**DOI:** 10.3389/fimmu.2020.610131

**Published:** 2021-01-22

**Authors:** Geoffrey P. Dobson, Erik Biros, Hayley L. Letson, Jodie L. Morris

**Affiliations:** Heart, Trauma and Sepsis Research Laboratory, College of Medicine and Dentistry, James Cook University, Townsville, QLD, Australia

**Keywords:** coronavirus, inflammation, trauma, infection, immune system, evolution

## Abstract

We present a brief history of the immune response and show that Metchnikoff’s theory of inflammation and phagocytotic defense was largely ignored in the 20^th^ century. For decades, the immune response was believed to be triggered centrally, until Lafferty and Cunningham proposed the initiating signal came from the tissues. This shift opened the way for Janeway’s pattern recognition receptor theory, and Matzinger’s danger model. All models failed to appreciate that without inflammation, there can be no immune response. The situation changed in the 1990s when cytokine biology was rapidly advancing, and the immune system’s role expanded from host defense, to the maintenance of host health. An inflammatory environment, produced by immune cells themselves, was now recognized as mandatory for their attack, removal and repair functions after an infection or injury. We explore the cellular programs of the immune response, and the role played by cytokines and other mediators to tailor the right response, at the right time. Normally, the immune response is robust, self-limiting and restorative. However, when the antigen load or trauma exceeds the body’s internal tolerances, as witnessed in some COVID-19 patients, excessive inflammation can lead to increased sympathetic outflows, cardiac dysfunction, coagulopathy, endothelial and metabolic dysfunction, multiple organ failure and death. Currently, there are few drug therapies to reduce excessive inflammation and immune dysfunction. We have been developing an intravenous (IV) fluid therapy comprising adenosine, lidocaine and Mg^2+^ (ALM) that confers a survival advantage by preventing excessive inflammation initiated by sepsis, endotoxemia and sterile trauma. The multi-pronged protection appears to be unique and may provide a tool to examine the intersection points in the immune response to infection or injury, and possible ways to prevent secondary tissue damage, such as that reported in patients with COVID-19.

## Living Systems: The Steady-State and Design Tolerances

A superficial consideration of the totality of the processes in the living being immediately shows that in the strictest sense a dynamic equilibrium is never present.

E. Pfluger (1877) (1) p57

Living systems are not equilibrium states; they are steady-states requiring a continual flow of matter, energy and exchange with the environment (2, 3). The concept of a steady-state began in 1877 with Pfluger’s “*natural adjustments*”, Bernard’s concept of “*milieu intérieur*” (1878), and Richet’s “*living beings were stable but modifiable*” (1900) (3, 4). It was not until the early 1920s that Walter Cannon combined these ideas into a unified scheme of homeostasis (5). Cannon argued that the living organism was in a *dynamic state of constancy*, with its constituent parts and processes being actively maintained in balance despite external fluctuations. He believed homeostasis was a systems phenomenon: “peculiar to living beings - involving, as they may, the brain and nerves, the heart, lungs, kidneys and spleen, all working cooperatively” (5). The addition of negative and positive feedback circuits to support homeostasis was not Cannon’s idea, but entered in the mid-1930s from Russian physiologist Pyotr Anokhin’s theory of functional systems (6). The steady-state was now viewed as comprising the sum of negative and positive feedback mechanisms that maintains a living system within a range of operational limits or tolerances. *A stress, injury or infection was a challenge to the body’s steady-state, and the major goal of any new drug therapy, device or intervention is to restore homeostatic balance*.

## Exceeding The Body’s Defense Capability After A Barrier Breach

Except on few occasions, the patient appears to die from the body’s response to infection rather than from it.

Sir William Osler (1904)

During the life of an organism, the immune system continually senses and responds to barrier breaches and threats. If a breach occurs, the number of blood-borne and tissue-resident immune cells can change dramatically in seconds (7, 8). “Tissue residency” refers to immune cells that already reside in the tissue parenchyma or stroma, where they can roam freely without moving from tissue to tissue (9). A breach is defined as a break in epithelial continuity, external or internal, that may arise from a pathogen (e.g. viruses, bacteria, fungi, protozoa, or helminth) or sterile injury. Sterile injury is defined as a trauma in the absence of pathogen. However, trauma is rarely sterile and can be colonized by opportunistic pathogens, soon after a penetrating injury, which may lead to secondary infection. From our hunter-gatherer, protohuman origins, the body has developed, through natural selection, a defense system against infection or injury that is normally robust, self-limiting and restorative (4, 10, 11). The immune response normally neutralizes a pathogen or promotes wound healing. *However, if the threat overwhelms the body’s internal tolerances, such as witnessed in some patients during the current COVD-19 pandemic or after major trauma, excessive sympathetic outflows, inflammation, coagulopathy, endothelial and metabolic dysfunction can occur leading to multiple organ failure and death* (11, 12). Currently, it is not known, for example, why COVID-19 triggers such an explosive inflammatory response in some patients, and not in others (13–16). Understanding the mechanisms responsible for these different responses resides in the control of the immune system (17).

In this review we will: 1) present a brief history of the host’s immune response to infection or trauma, 2) discuss the importance of inflammation and underlying molecular defense mechanisms in the context of an infectious diseases like COVID-19, and 3) discuss how a new therapeutic approach using adenosine, lidocaine and magnesium (ALM) may alter the host’s phenotype to prevent or resolve hyperinflammation, and help return the system to “normal” operating conditions. We address the following questions:

• How does the host mount an immune defence against a pathogen?

• Is the 20^th^ century self/nonself discrimination still a useful concept?

• How does the host discriminate infection from sterile injury?

• What role does local and systemic inflammation play in host defence?

• Are there common intersection or checkpoint points that could lead to new drug development to bolster the hosts’ immune defence against infection and sterile injury?

## Brief History of Immune Defense: From Macrophages to Clonal Theory

When I first put forward the biological theory of inflammation eight years ago, I expressed the idea that this reaction is effected by the intermediation of a physiological continuity between “the cells of the connective tissue, those of the endothelial wall and the leucocytes, which form a complete chain and play the principal part in the inflammation of vertebrates.” The connective tissue cells which are first attacked, would, I thought, transmit the action to the vascular wall, the cells of which would contract to facilitate the passage of the white corpuscles.

E. Metchnikoff (1893) (18) p191

## Origins of Cell and Humoral Theories of Immunity

Around 130 years ago, Russian zoologist and pathologist Elie Metchnikoff (1845–1916) was among the first to develop a cellular theory of inflammation and phagocytic defense against pathogens (18). The theory was built on Rudolf Virchow’s cellular basis of disease (19), and Pasteur’s pioneering research on vaccine development (20). For his model, Metchnikoff embraced Darwin’s laws of natural selection, and although he was not the first to observe phagocytosis and inflammation, he does appear to be the first to highlight the importance of blood-borne and tissue-resident macrophages (21, 22). Metchnikoff viewed inflammation as a highly integrative and restorative process (23). He also intuitively drew parallels between phagocytes devouring the tadpole’s tail, which was “eaten” at the appropriate time of metamorphosis, to wound repair and bacterial killing from amoeba to humans (23). As a sideline, but related, Metchnikoff anticipated the importance of the gut “microbiome” to immune daily health by eating yogurt, or other types of sour milk, to cultivate beneficial bacteria for host health (24).

Opponents of Metchnikoff’s cellular scheme advocated the antibody (or “antitoxin”) theory of immunity, which became more popular. The new movement was largely driven by Emil von Behring and Japanese bacteriologist Shibasaburo Kitasato, who argued that antibodies provide greater specificity to ward off foreign invaders than a freely mobile phagocyte in an inflammatory environment (25, 26). They showed “serum therapies” extracted from the blood of naturally or artificially immunized animals induced immunity in sick patients suffering diphtheria or tetanus, a practice used today in some COVID pandemic patients (27). The partial success of serum therapies, and later experiments in mice, led German immunochemist Paul Ehrlich to develop his “side-chain theory” of immunity. Ehrlich believed that living cells were covered with chemical side-chains that formed links with foreign toxins, and when under threat, the cell would produce more side-chains to bind the toxin (or antigen) (26). For every antigen in nature, there is at least one side-chain that will bind it, and once detected, more cells can be made with the same side-chain, which are released into the blood as “antibodies”, in a feed-forward manner. *Over time, Ehrlich believed that the host builds a “memory” immunity “ready-made” in their blood to protect against subsequent exposures to the same infection*. Despite its ingenious specificity and refinement, Ehrlich’s humoral hypothesis had a number of shortcomings, as it did not explain why some cells possessed the ability to make side-chains, and others did not (28). Metchnikoff and Ehrlich shared the Nobel prize in 1908 for their different theories, and the modern era of immunology was born (29).

## Clonal Selection Theory of Immunity: One Cell Makes One Antibody

Ehrlich’s idea that antibodies were already present in blood was also rejected in 1930 by Fritz Breinl and Felix Haurowitz (28, 30). In its place, they developed the “template instructive hypothesis” of immunity, which was later adopted by Linus Pauling (28, 30, 31). The theory proposed that foreign antigens served as “templates” of antibody globulin production, and helped to explain how Karl Landsteiner could stimulate the formation of antibodies from *artificially generated substances*, known as haptens (28). Although popular, the “template” theory did not explain a number of critical experiments that showed humans at birth *already had a “pre-immune” antibody repertoire (in absence of antigen)*, or why antibody production during one’s life was enhanced *after a second inoculation* (adjuvant) with faster, stronger and long-lasting immunity (30). In 1955, Danish immunologist Niels Jerne proposed an alternative natural selection theory of antibody formation, where he argued that antibody diversity was part of the host’s *“in-built” memory*, which anticipated antigenic interactions rather than being a consequence of antigen exposure (31–33). This theory was a game-changer because it moved the focus of immunity from the *antigen-antibody response* to the host’s *antibody-producing cells* themselves, which were known to display memory-like functions (23).

**Figure 1:**
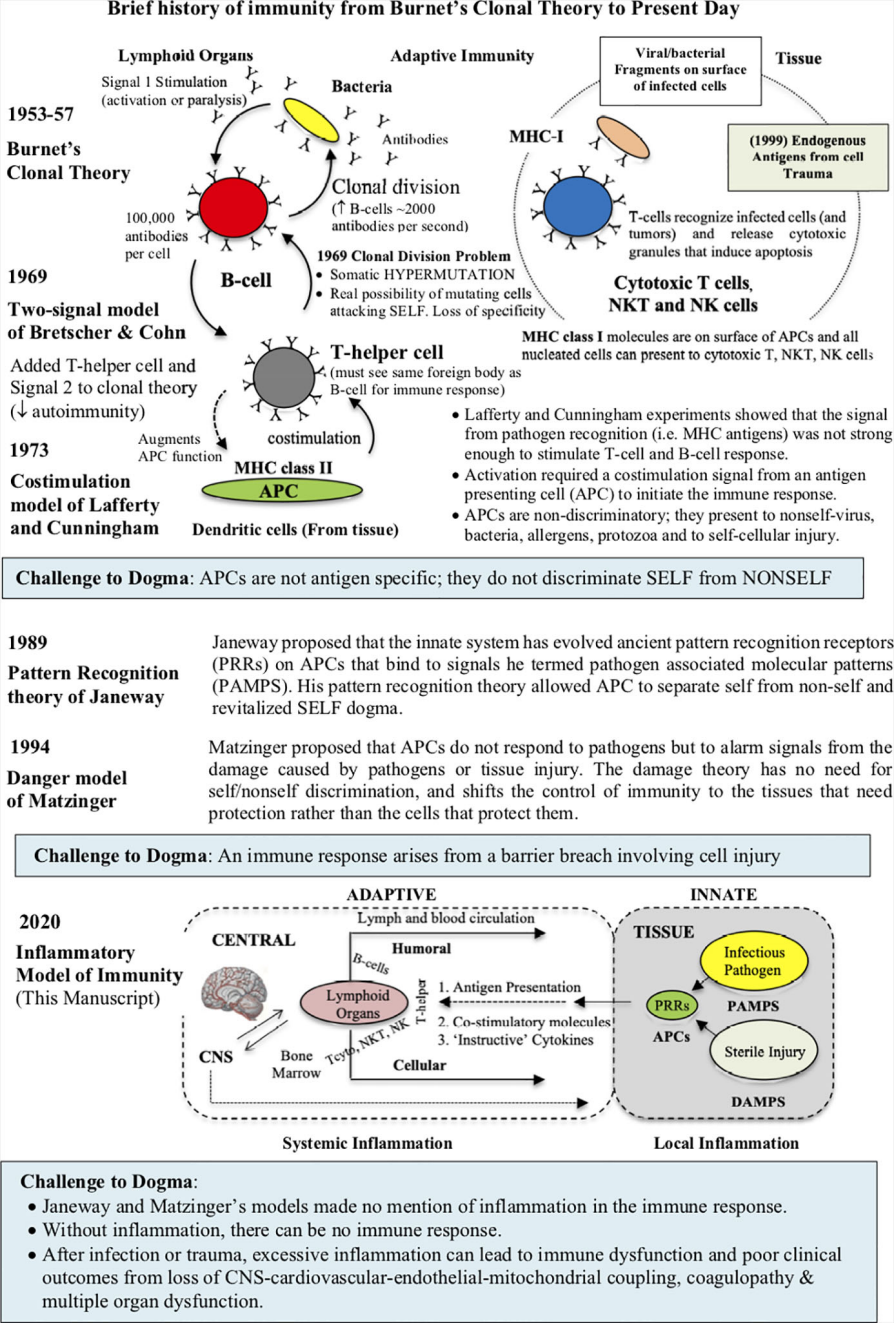
Brief history of the working models of immunity from Burnet’s clonal theory, Bretscher and Cohn two-signal model, Janeway’s pattern recognition receptor (PRR) theory, and Matzinger’s danger theory. In 1989, Janeway’s model, building on the earlier work of Lafferty and Cunningham, ingeniously relegated the innate function of antigen presenting cells (APCs), that roam the tissues, to recognize highly conserved microbial components of ancient origin. He called these microbial components pathogen-associated molecular patterns (PAMPS), which serve as ligands for a broad class of PRRs located on innate cells. While Matzinger’s danger theory recognizes Janeway’s contribution, APCs are more responsive to cellular damage, irrespective of the presence of pathogen or sterile injury (DAMPS). Despite their different individual contributions, no model is complete as they fail to appreciate the major contribution of inflammation. An inflammatory model has been added to the current theories of immunity (see text for more information). Depending upon the type and location of antigen, DCs can activate two arms of adaptive immunity; humoral activation (B cell *via* MHC class II molecules) from extracellular pathogens and toxins, and cell-mediated immunity (cytotoxic T cells, NKT, NK and macrophages *via* MHC class 1 molecules) from intracellular pathogens or tumors.

In the late 1950s, Jerne’s work stimulated immunologists David Talmage and Frank Macfarlane Burnet to propose two cell selection theories (32). According to both theories, a foreign “antigen” binds to the host’s antigen-specific lymphocyte, generates a signal, and activates the lymphocyte to rapidly divide and make exact copies (26, 32) (**Figure 1**). While sharing some similarities, Burnet’s clonal theory explained in great detail: 1) why there was such a high probability of warding off a foreign attack from a lymphocyte recognizing one epitope and dividing, and 2) how “self” was protected from attack because it was developed early during embryogenesis in an environment where there was no external threats (26, 32). According to Burnet, it was only *after birth*, beginning with a clean slate, that an individual’s immune response discriminated self from non-self (32, 34, 35). During lymphocyte development, the embryo removed or inactivated any autoreactive clones*, and the remaining lymphocytes only become responsive after birth*. Burnet’s idea for germline selection, and the specificity that one lymphocyte makes one antibody, was later supported by the studies of Peter Medawar and Gus Nossal (26, 32). Burnet’s separation of the germline selection theory (innate) and adaptive (during life) immunity meant that the latter can respond to millions of different foreign antigens in a highly specific way, without causing harm to the host. Today, the adaptive immune system comprises humoral immune responses, orchestrated by B-lymphocytes (B cells), and cell-mediated immune responses, orchestrated by cellular T-lymphocytes (T cells), that are developed successively over a lifetime (**Figure 1**). The adaptive immune responses are distinct from innate immunity, since they involve specificity and immunological memory (36).

## Post-Clonal Theory Era: Signaling was a Problem

Despite its simplicity and elegance, Burnet’s clonal model had a number of limitations. In the 1960s, the first big unknown was what kind of signal was generated to initiate an immune response, and how was it presented to the host, and subsequently processed (37). On the question of antigen presentation, Bretscher and Cohn argued on theoretical grounds that Burnet’s one signal-one response lacked specificity [38, 39 Bretscher, 2019 #5959]. They argued, given the tens of billions of circulating cells in blood, one signal increased the likelihood of a lymphocyte hypermutation that could attack “self” (37–39). In its place, they suggested two signals; one by the receptor antibody of the B cell (Signal 1), and a second ‘partner’ signal by T-helper lymphocytes, that recognized the same antigen (Signal 2) (**Figure 1**) (37, 40). *The idea that lymphocyte activation required lymphocyte cooperation provided a check-point over mistaken identity and untoward antigenic activation* (37). Bretscher and Cohn further suggested that the second “partner” signal may involve a membrane/membrane interaction between the two interacting cells (37). Today, this two-signal hypothesis has been experimentally verified; B-cell activation requires interaction with a specific ligand (CD40), which is expressed on the surface of an activated helper T cell (CD4^+^ T cells) (37, 41, 42).

The next major advance to antigenic signaling came from studies of Lafferty and Cunningham who showed that the two-signal model suffered from submaximal activation, and required some form of booster ‘signal’ (43, 44). *The antigen to antibody signal (even with T-helpers) was too weak to stimulate an immune response*. They proposed a costimulation signal, which they believed must come from another cell or cells located in the peripheral tissues, which they termed antigen presenting cells (APCs) (44). In 1973, the dendritic cell (DC) was the first APC discovered by Canadian physician Steinman and Cohn in lymph nodes and spleen (45) (**Figure 1**). Mature macrophages, and other tissue-resident immune cells, also have this antigen presenting capacity, though not to the extent of DCs (46–49). *The “costimulatory” APC signal of Lafferty and Cunningham was an on/off signal, and spatially separated from the “priming” or “helper” signal of Bretscher and Cohn*. In addition, shifting the initiating signal from the central lymphoid tissues to the periphery directly challenged the concept of self/nonself discrimination, since APCs cannot differentiate between the two (23, 50). These were exciting times for the field of immunology.

## Janeway’s Pattern Recognition Receptor Theory: Relegating Innate Immunity to Ancient Times

I will argue that the solution to both problems (diversity and specificity) existed prior to the development of the adaptive immune response and persists in contemporary mammalian immune systems.

C. Janeway (1992) (51) p11

The apparent irrelevance of self/nonself discrimination by APCs captivated immunologist Charles Janeway in 1989 and challenged him to rethink the problem. Janeway began by exposing the “immunologist’s dirty little secret”, namely, that foreign antigen alone was insufficient to elicit the adaptive immune response (52–54). He further questioned why T helper cells were required to activate B and T cells, and yet foreign antigen could either activate or inactivate the same cells *in the absence of T helper cells*. Janeway’s basic assumptions for his scheme are listed in **Table 1**. After exposing the weaknesses of prevailing models, Janeway ingeniously postulated that costimulation was only switched ‘on’ if the host’s APC’s possessed specific pattern recognition receptors (PRRs) that recognized some common pathogen‐associated molecular pattern (PAMP) from an invading pathogen (54) (**Figure 1**).

**Table 1:** Major assumptions that led Charles Janeway to develop his pattern recognition receptor (PRR) theory of innate and adaptive immunity.

Innate immunity: present in invertebrates and vertebrates
1) Acts as a first responder to infectious pathogens.
2) Recognizes antigens on pathogens via PRRs “that are ancient in their lineage”.
3) PRRs are located on tissue-resident APCs.
4) If not resolved locally, an adaptive immune response is activated.
**Adaptive immunity: unique to vertebrates**
5) Slower to respond and involves B and T cells.
6) APCs communicate to B and T cell receptors via PRRs.
7) Two distinct types of antigen presentation (MHC class I/MHC class II).
8) Mature T cells become activated and begin dividing rapidly by mitosis (clonal expansion) to amplify the response to effectively control a pathogen.
9) Two advantages: Specifically tailored to the invading pathogen: Each cell is committed to make one antigen-specific receptor protein, and the host’s system can respond to many pathogens (>100 trillion). Forms a pool of memory cells from these specific effectors that can last for many years.
10) Repeat infections can build the host’s immunity, in most cases.

APC, antigen presenting cell; MHC, major histocompatibility complex.

Janeway’s concept of PRRs has been experimentally supported with a long list of PAMPS, which led him, and colleague Ruslan Medzhitov, to clone the first human Toll-like receptor (TLR). Together, they showed that TLR stimulation activated signaling pathways required for the development of adaptive immunity (55, 56). This demonstration provided strong support on the significance of TLRs, and their ligands, to initiate an immune response (57). Janeway and Medzhitov further proposed that host innate recognition had ancient roots, and if PAMPS were not present or detected, no immune response would occur. *Key to Janeway’s thinking was that the host’s innate system was part of the germline selection process, that APCs had evolved receptors that recognize infectious bacteria or their components, and that they were evolutionary distinct from clonal receptors derived from adaptive immunity after birth* (52, 55). Here, the remnants of Burnett’s clonal theory are apparent, with modifications driven by intuition and experiment. Moreover, since PAMPS are foreign, and not produced by the host, the immune system can efficiently discriminate self from nonself, and instruct the adaptive immune system to respond accordingly (51). Janeway and Medzhitov believed they had solved this fundamental problem posed by Lafferty and Cunningham having with regard to APC surveillance in the periphery. Much debate exists as to why Janeway and Medzhitov were not awarded the 2011 Nobel Prize for their unification theory, and TLR signaling pathway demonstrations (57). The prize was shared between Jules Hoffman (described Toll genes in fruit flies, 1996), Bruce Beutler (showed LPS activated TLR-4 receptor, 1998) and Ralph Steinman (discovered DCs, 1973) for their discoveries on innate immunity (57).

## Matzinger’s Danger Model: Moving Beyond the Immune Self/Nonself Dichotomy

Although this (self/nonself) paradigm has often served us well, years of detailed examination have revealed a number of inherent problems. This viewpoint outlines a model of immunity based on the idea that the immune system is more concerned with entities that do damage than with those that are foreign.

P. Matzinger (2002) (58) p301

In 1994, Polly Matzinger challenged Janeway’s theory and his PAMPS self/nonself dichotomy. She proposed the primary function of the immune system “is the need to detect and protect against danger” (59). Notwithstanding the special case of foreign antigen recognition, Matzinger argued the host’s innate immune cells must also recognize molecular signals from cellular damage (59). Matzinger termed these danger or alarm signals, damage-associated molecular patterns (DAMPs) (35, 39, 59, 60). Danger or alarm signals arise from cellular damage caused by pathogens *or* tissue trauma. Up until now tissue injury had received very little attention, and Matzinger’s views were intuitive and transforming. *She proposed that DAMPS and PAMPS share the common property of signaling cellular damage, irrespective of its origin*. The danger theory also challenged Janeway’s concept of “foreign”, meaning the host’s infectious “nonself” (**Figure 1**). Vertebrates have evolved harmoniously with many billions of friendly “foreign” microbes living in their intestines, mouth and epithelial surfaces without causing harm. Indeed, animals and humans have evolved two genomes; their own and the gut microbiome (61, 62). Today, we know there are more bacteria residing in our gut (10^15^) than cells in our body (10^14^), which are not infectious *unless there is a barrier breach* (39, 60, 61). Every day we inhale hundreds of thousands of bacteria per cubic meter of air, and many of these inhabit our nasal-pharyngeal passages (63). These friendly “foreigners” contribute to our immune health as part of the gut–brain–immune axis (64), and have the potential to become pathogenic when a breach occurs from ischemia, trauma or disease (62).

Conceptually and operationally, Matzinger’s theory shifted the molecular specificity of innate activation to damage in the tissues. While sharing many features of Janeway’s PRR theory, Matzinger concluded there was no need for a self/nonself binary distinction to explain immunity. She wrote: “to say that specificity is important is different from saying that a discrimination between self and nonself is necessary” (39). The immune response was now viewed as a response to tissue damage or alarm “signals” that don’t discriminate (60). Over the decades, Matzinger’s danger model has received a great deal of experimental support, with a long list of DAMPs from damaged or dying cells (35, 58, 60, 65). Despite their differences, Matzinger and Janeway’s theories largely focused on the early molecular specificity of the innate immune response, and not the regulation of adaptive immunity (66).

## Inflammation: A Missing Piece of the Puzzle

Our understanding of inflammation started with research in the field of leukocyte migration, meticulously observed under the microscope by Metchnikoff and colleagues. … He can be considered the father of innate immunity.

B. Imhof (2016) (22) p655

From an historical perspective, it is remarkable that the models of immunity rarely included Metchnikoff’s theory of inflammation and phagocytotic defense (67). There was no mention of ‘inflammation’ in any of the key papers of Janeway and Matzinger (51, 59), or in later reviews on the “danger theory; 20 years later” (35). To be fair, cytokine biology was in its infancy and the first drugs targeting the inflammatory system were not developed until the 1990s (68). However, given that cytokines were known to be produced by lymphocytes and macrophages in the 1970s (69), and Lafferty and Cunningham had shifted the initiating immune signal from lymphoid tissues to the periphery (1975), it is still curious why there was no eureka moment, at least in their writings, linking the innate immune response to inflammation, and recognizing that immune cells were inflammatory cells. It appears that Janeway and Matzinger were more interested in molecular aspects of the initiating signals and the concept of self/nonself discrimination, rather than immune cell interactions and the changing milieu underpinning this response.

**Figure 2:**
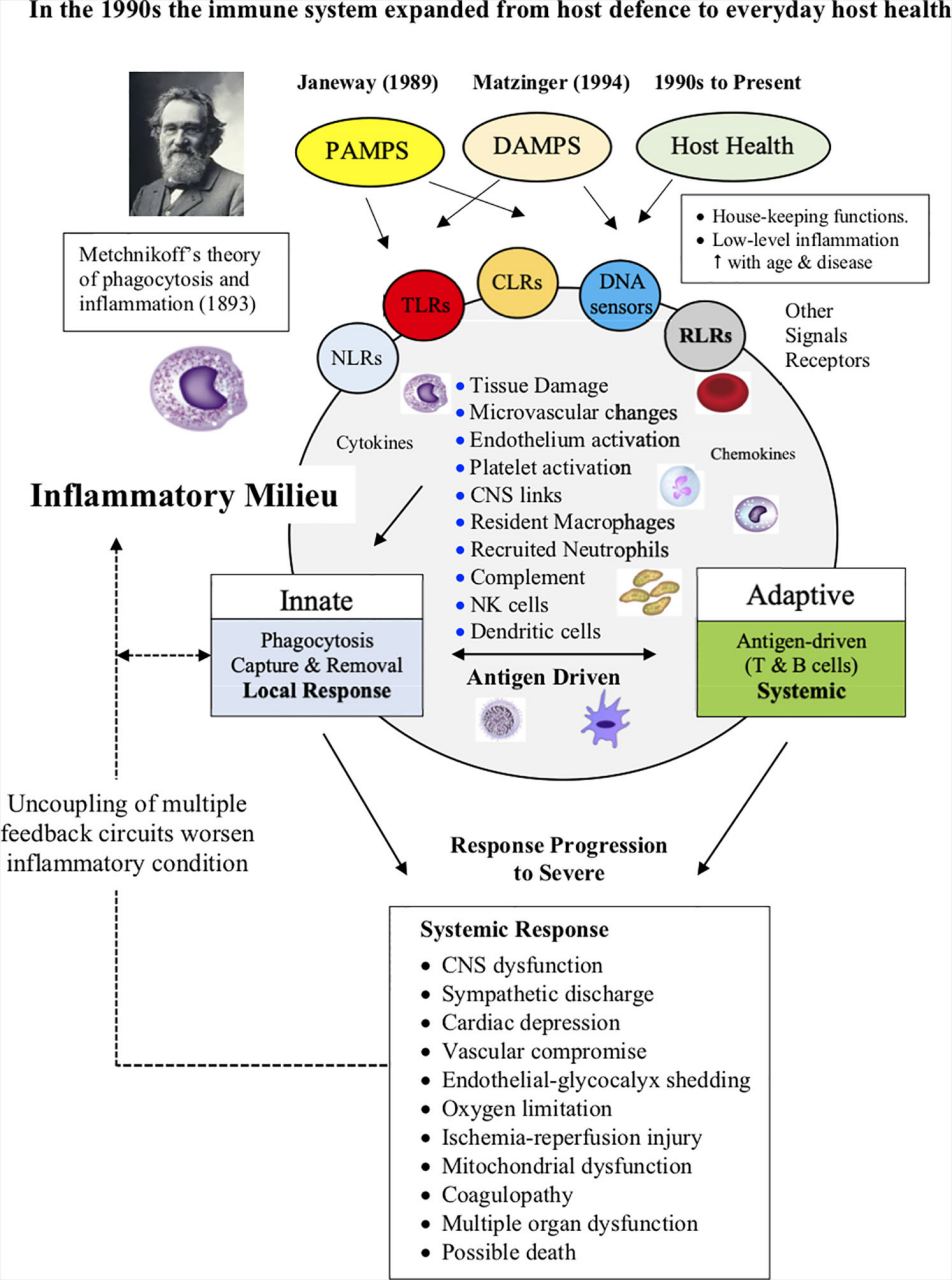
Without inflammation there is no immune response. Changes in the inflammatory milieu and low oxygen drives the type and extent of the immune response. Metchnikoff’s theory of inflammation and phagocytotic defense was largely ignored throughout the 20^th^ century. In the 1990s, the role of the immune system expanded from defense to maintaining host health, which includes a low-level of inflammation associated with general housekeeping functions and maintenance of whole body steady-state. The adaptive response proceeds only if there is sufficient antigen to drive the process. In severe cases, inflammation can spread to become a systemic response and activate a CNS-sympathetically-driven “fight-or-flight” stress response, and if not controlled, can escalate into widespread immune, cardiovascular and metabolic dysfunction, multiple organ failure and death.

Like most paradigm shifts in science, it is difficult to pin-point when inflammation entered the different models of immunity. It appears to have occurred in the 1990s when cytokine biology and technologies were rapidly advancing. For example, in 1993 Ferrara reported an explosive inflammatory response with profound immune dysregulation in patients receiving allogenic tissue graft transplantation (70, 71). Ferrara coined the term “cytokine storm” to describe this response, whereby donor T cells produce excessive quantities of proinflammatory cytokines that induced damage and pathology in the recipient’s tissues (70). A few years earlier, Beutler and colleagues showed that cytokines, such as IL-1, TNF-α, and IL-6, played a direct role in the pathogenesis of endotoxic shock (72), and Cavaillon showed experimentally that individual immune cells possessed the ability to produce their own source of regulatory cytokines that modulated their effector functions (69, 73). There are many other examples showing that cytokines, inflammation, immune function and disease were increasingly being recognized as part of the same intertwined process. Another aspect not widely reported until the 1990s, is that the main role of the immune system expanded from host defense to maintenance of host health (**Figure 2**). This new role involved a “low-level” innate inflammatory response with housekeeping functions such as removal of stressed or aged cells and replacing them with new ones, maintaining symbiotic exchanges with the gut microbiome, maintaining CNS-cardiac health *via* multiple feedback networks, integrating inter-organ exchange, endothelial health, control of the stress response *via* shared receptors and hormones, restoring the steady-state after trauma, wound healing, and preventing chronic diseases, including autoimmune diseases and cancer (**Figure 2**).

Control of inflammation is key for immune defence and everyday host health. Without inflammation there is no immune response.

## Inflammation: A Brief History

The Roman Celsus is credited as first documenting (1st century AD) the four cardinal signs of inflammation: *rubor et tumor cum calore et dolore* (redness and swelling with heat and pain).

Scott and colleagues (2004) (74)

The term *inflammation* is derived from Latin *inflammare (to set on fire)* (74, 75). Before the 18^th^ century, acute inflammation was regarded more as a disease, involving Celsus’ redness, pain, heat and swelling, and a surcharge of blood in the tissues (74). In the third century AD, Galen also believed that inflammation was the body’s reaction to an injury, an idea taken up in the mid-1700s by surgeon and anatomist John Hunter (1728–1793). “This inflammation”, Hunter wrote, “will generally be in proportion to the degree of injury done, the nature of the parts injured, and the state of the constitution at the time” (76) p243. Hunter also appreciated that inflammation was universal, beneficial and restorative: “But from whatever cause inflammation arises, it appears to nearly the same in all, for it is an effect intended to bring about a reinstatement of the parts nearly to their natural functions” (76) p286. In the mid-19^th^ century, Virchow viewed inflammation as inherently pathological, and in the second half of the century, as changing cell populations in blood and the tissues, which provided a backdrop to Metchnikoff’s phagocytosis theory (74).

**Figure 3:**
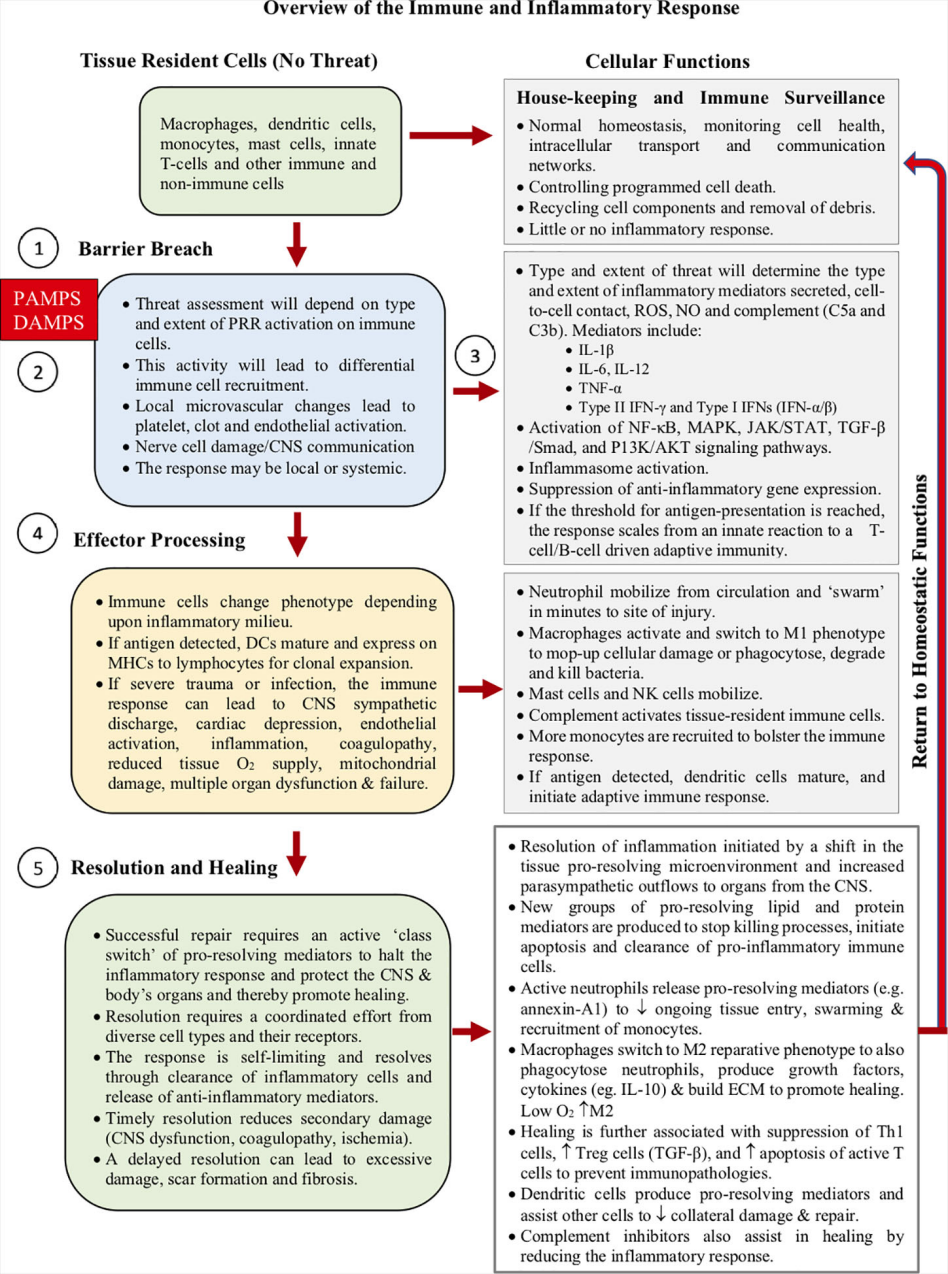
Overview of the immune response. The time-course depends upon the precise nature and severity of the initial threat. The process comprises five main components; 1) initiator signals **(**DAMPS and PAMPS), 2) tissue-resident “innate” cells, and their receptors (PRRs), 3) cell signal transduction pathways, 4) effector responses to neutralize the threat (innate and adaptive), and 5) active resolution to promote healing. Inflammation is key to the selection, recruitment, and phenotypic expression of the various immune cells during the immune response. How damage control is communicated among immune cells, and their subsequent go/no-go “decision-making criteria”, to start healing with epithelial closure, are not well understood.

Today, a typical inflammatory response consists of at least five components (77–81) (**Figure 3**):

1. Initiating signals: DAMPS and PAMPS
2. Innate pattern recognition receptors: initial sensing of DAMPS and PAMPS
3. Cell signaling transduction pathways
4. Effector responses to remove the threat (local and systemic)
5. Resolution or containment strategies to promote healing

Below, we discuss the five components of inflammation and the linkages and possible intersection points that activate the process, and its timely resolution for optimal healing.

### 1) Initiating Signals: DAMPS and PAMPS

The innate immune response resides with the tissue resident macrophages (82), DCs (47), a subset of B memory cells (83), and mast cells detecting a barrier breach (84). Some T cells of ancient origins are also resident in tissues and include memory (T_RM_) cells, intraepithelial lymphocyte (IELs), invariant natural killer (iNKT) cells, and gamma-delta T-cell subsets (γδT cells) (85–87). These cells detect a myriad of molecular stimuli, the DAMPS and PAMPS, which comprise a complex mixture of proteins, lipoproteins, nucleic acids and saccharides (65, 88, 89). As mentioned above, DAMPS are released from damaged, stressed or dying cells, including extracellular and cell membrane, cytosolic, cytoskeleton, nuclear mitochondrial, vascular endothelial, and blood components (65, 90).

Some DAMPS include fibrinogen, annexins, platelet components, fibronectin, S100 proteins, syndecan-1, F-actin, ATP, histones, DNA, TFAM, mitoROS, cytochrome C, IL-1α, HMGB1, heparan sulfate, tenascin C, defensins, amyloid-β, and many others (90). PAMPS, on the other hand, are molecular signals from pathogens, or their components, that may directly or indirectly breach barriers such as skin, lung epithelium, or the lining of the gut, or from tissue injury that becomes infected. PAMPs can be derived from viruses, bacteria, fungi, and protozoa and helminths. Examples include double-stranded RNA from viruses, LPS from gram-negative bacteria, flagellin products from bacteria, DNA from pathogens, surface glycoproteins, lipoproteins, and other membrane components (53, 55, 65). *Importantly, DAMPS and PAMPS are not mutually exclusive and may share co-receptors and accessory molecules, and form “partnerships” to coordinate a response* (91). Antigens, key drivers of adaptive immune responses, are not normally considered PAMPs, and largely comprise proteins or polysaccharides, although small molecules coupled to carrier proteins can also be antigenic (e.g. haptens).

### 2) Innate Pattern Recognition Receptors: Initial Sensing of DAMPS and PAMPS

The LPS sensing role of TLR4 was a huge surprise.

Bruce Beutler (Nobel Laureate) Quoted from Ravindran (2013) (92)

DAMPS and PAMPs are detected by Janeway’s PRRs located on immune cells and non-immune cell types (e.g. astrocytes, neurones, cardiomyocytes, hepatocytes, gut and muscle cells) (93, 94). PRRs serve as “sensors” to communicate the nature and severity of the breach (94). Most, if not all, cells express at least five families of PRRs, including toll-like receptors (TLRs), RIG-I-like receptors (RLRs), NOD-like receptors (NLRs), C-type lectin-like receptors (CLRs), and cytosolic DNA sensors (95–97). These different PRRs are located either on the cell surface or intracellularly. TLRs are the best studied, and in humans, ten different motifs have been characterized (96–99). Structurally, TLRs are integral glycoproteins characterized by an extracellular ligand-binding domain containing leucine-rich repeat motifs, and a cytoplasmic signaling Toll/IL-1 receptor homology (TIR) domain (100). Ligand binding to TLRs through PAMP or DAMPS induces receptor oligomerization, which subsequently triggers intracellular signal transduction (90, 101). For example, TLRs can mediate cellular responses to bacterial LPS (TLR4), lipopeptides (TLRs 1, 2, and 6), flagellum (TLR5), and microbial RNA and DNA nucleotide sequences (TLRs 3, 7, 8, and 9) (102). As mentioned, TLRs are also expressed in most tissue cells. The most important immune cell types expressing TLRs are macrophages, DCs, mast cells and B cells (100). TLR9, for example, can be modulated on cardiomyocytes to reduce myocardial ischemia/reperfusion injury (101), and are believed to be involved in normal cardiovascular function and disease (103). Another important PRR that senses a wide range of PAMPS and DAMPS is the NLRP3 protein that activates a cytoplasmic multiprotein platform assembly known as the inflammasome (104). The inflammasome amplifies the inflammatory response *via* caspase-1 activation and IL-1β and IL-18 maturation, and is critical for host defenses against bacterial, fungal, viral infections and trauma (105, 106).

### 3) Cell Signaling Transduction Pathways: Linking Receptor Activation to Gene Expression

**Figure 4:**
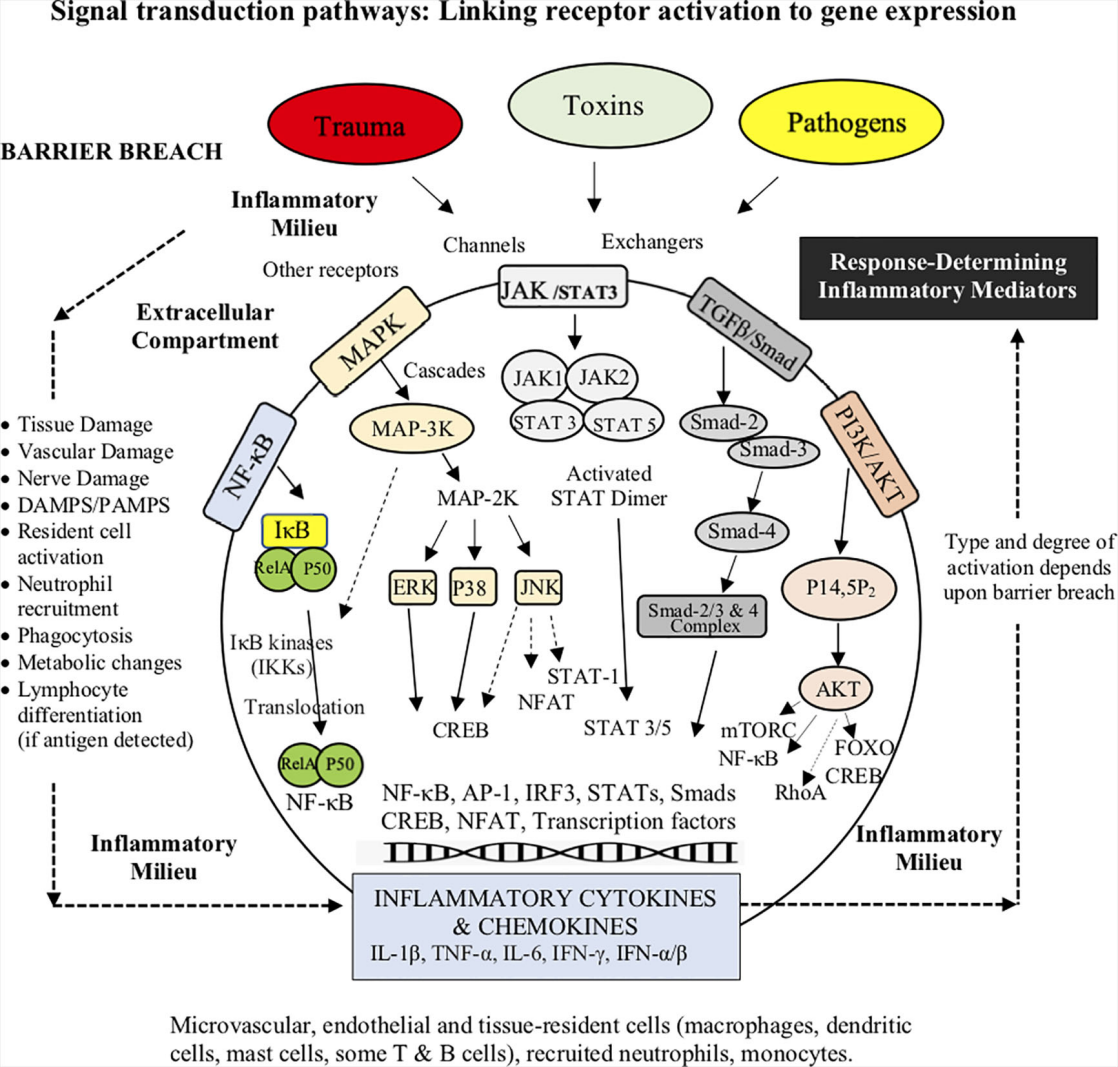
Broad schematic of the main signaling transduction pathways and the changing role of the inflammatory environment. The link between receptor activation and control of inflammatory cytokine and chemokine production involves at least five signaling transduction pathways; NF-κB, MAPK, JAK/STAT3, TGF-β/Smad and the PI3K. The cytokine TGFβ1 can also regulate NFκB and JNK pathways *via* TRAF6 convergence. Although the five major pathways are regulated by different mechanisms, they share many PRRs, stressor signals (e.g. adrenergic, cholinergic, receptors/ion channels) and accessory mediators (e.g. growth factors, hormones), and converge at common intersections points, such as NF-κB, AP-1 and the CREB family. NFκB signaling (not shown) also participates in the regulation of the NLRP3 inflammasome, which is involved in the rapid amplification of inflammation and its resolution.

Once PRRs are activated, the intracellular pathways that modulate the inflammatory response involve a network of cascades and interconnections. There are at least five major signaling transduction pathways; NF-κB, MAPK, JAK/STAT3, TGF-β/Smad and PI3K/AKT (**Figure 4**) (88, 94, 107–111). If TLRs are activated, different signals may be directed into two distinct pathways with different cytokine products: 1) the myeloid differentiation primary response protein 88 (MyD88)-dependent pathways, which is activated by most TLRs and IFN-γ, or 2) the TIR domain-containing adaptor-inducing IFN-β (TRIF)-dependent pathway, which responds to only a few TLRs, such as TLR3 and TLR4 (111, 112). If a robust innate immune response is required, both MyD88 and TRIF pathways can be activated (113), which leads to activation of three transcriptional factors, NF-κB, activating protein-1 (AP-1) and IFN regulatory factor 3 (IRF3) (114–116) (**Figure 4**). Similarly, “booster” coactivation of NF-κB and AP-1 can occur *via* the MAPK pathway (117), and additionally FOXO and cAMP Response Element Binding (CREB), if the phosphatidylinositol-3-kinase (PI3K/AKT) pathway is triggered (118) (**Figure 4**). Other transcriptional factors STAT3, p53, estrogen receptor, ATF3 (CREB family), Smad3 and 4, and NFATs all have the ability to intersect and regulate NF-κB (115, 119). Possible reasons for why NF-κB was selected as a principal regulator of inflammation, and check-point for multiple pathway and crosstalk interactions, may be to expand gene expression in a more robust, controlled manner to deal with threats of diverse origins. *From an evolutionary perspective, having multiple networks to finely tune the inflammatory response in a context- and time-specific manner may have conferred the host a major survival advantage to a pathogen or injury.* Single pathway activation may not have been sufficient to induce an adequate effector response (113).

The ultimate goal of PRR signaling is to change immune cell activation states, and production of inflammatory mediators (107). For example, TLR stimulation *via* both MyD88 and TRIF signaling induces the maturation of DCs, specifically the upregulation of costimulatory molecules (e.g. CD40, CD80 and CD86) and the production of proinflammatory cytokines (e.g. IL-6, and TNF-α) (120). The main function of cytokines is intercellular communication among and between immune and non-immune cells as autocrine, paracrine, or endocrine messengers to modulate the host’s effector response (121, 122). Other inflammatory mediators include chemokines, vasoactive amines (histamine and serotonin), adhesion molecules, neurogenic receptors (e.g. dopamine, adrenergic, glutamate acetylcholine and serotonin), vasoactive peptides (Substance P), complement components (C3a, C4a and C5a), lipid mediators (leukotriene B4, prostaglandins and platelet-activating factors), regulators of the extracellular matrix, pro-resolving mediators (lipoxins, resolvins, protectins, maresins, adenosine), and proteolytic enzymes (elastin, cathepsins and matrix metalloproteinases) (77, 78, 121–126). Upregulation or downregulation of inflammatory gene expression is also influenced by changes in the intracellular environment such as calcium handling, pH, redox coupling, nitric oxide, hypoxia status, endosomal and lysosomal activity, glycolytic and mitochondrial energy metabolism, and the generation of reactive oxygen species (ROS) (115, 119, 127–131). Calcium handling and oxidative stress, for example, if not tightly regulated, can lead to pathway dysregulation, mitochondrial dysfunction and genomic instability (131). This often occurs after major trauma or infection and can lead to immunodeficiency, septic shock, or induction of autoimmunity (see below) (94). Orchestration and timing of production of these inflammatory mediators shapes the type and extent of acute immune response to a threat (107).

### 4) Effector Inflammatory Responses to Remove the Threat (Local and Systemic)

In acute inflammation, we find, as a general rule, vascular dilatation accompanied by an active condition of the endothelium of the vessel-walls and an exudation with diapedesis, that is to say, three events which concur in producing a considerable afflux of leucocytes towards the injured spot.

E. Metchnikoff (1893) (18) p171

The innate effector response can range from a local Metchnikoffian phagocytotic engulfing, digesting, debris clearance and tissue repair, with no further action, to a full-blown, centrally-coordinated, clonal expansion of lymphocytes, if the host becomes *overwhelmed* with antigen (**Figures 1** and **2**) (41). This innate strategy may apply to small wounds which are open to opportunistic bacterial or viral intrusion. Thus, if antigen has not reached threshold, it appears a pathogen threat can be dealt with locally without activating the adaptive immune response (132–134). While trauma *per se* does not activate adaptive immunity in a conventional manner, an increasing number of studies involving CNS and musculoskeletal injury, have shown that T cells and B cells can be activated by endogenous “antigens”, independent of a pathogen (135, 136). The endogenous “antigens” are believed to belong to a class of DAMPS that stimulate autoantibodies, that may assist in cellular defense and repair (136). However, like inflammation, where there is overactivation of T or B cells, there is potential for collateral tissue damage and detrimental autoimmunity (94). In the case of major trauma, the immune cell effector response can quickly become a CNS-sympathetically-driven “fight-or-flight” stress response, and if not controlled, can escalate into widespread cardiovascular and metabolic dysfunction, systemic inflammation, immunosuppression, multiple organ failure and death (11, 12, 135, 137–139).

At the breach site, irrespective of the type of threat, there is an increase in blood supply and a myriad of local signals that prime and instruct innate immune cells to respond. These signals are derived from activated vascular endothelial and tissue cells, leakage of serum components (component), damaged nerve cells, platelets, neutrophils, macrophages, mast cells, monocytes, natural killer (NK) cells and DCs (140–145). *These different immune and non-immune cells, through their cytokine networks, play pivotal roles both as producer cells and target effector cells to produce the right response*. Recruited neutrophils produce ROS to help them neutralize rogue/damaged cells *via* phagocytosis, degranulation and extracellular traps (109, 146). In this proinflammatory environment, macrophages also switch to a M1 killer phenotype (induced by IFN-γ or TNF-α) and with the help from mast cells, recruit more neutrophils from the circulation to swarm into the site (7, 146–149). M1 macrophages are also activated by complement receptors (C3a, C5a, and C5b) (independent of antibody), which can induce the activation of the NLRP3 inflammasome to amplify the inflammatory response (147, 150). Resident innate NK cells also secrete cytokines, such as IFN-γ and TNF-α, and interact with macrophages, and other immune cells, to enhance the response (151). As the attack progresses, more neutrophils swarm in and blood monocytes are recruited to resupply tissue macrophages (126, 152).

In the case of infection, the bridge between innate and adaptive immunity, and induction of classical immune memory in lymphocytes, begins at the tissues with the activation of resident DCs (via complement and cytokines) (36, 46, 47, 153). In peripheral tissues, DCs: 1) capture and process antigens, 2) express lymphocyte co-stimulatory molecules, 3) migrate to secondary lymphoid organs and 3) secrete “instructive” cytokines to initiate and drive lymphocyte differentiation and clonal expansion (**Figure 1**) (128). Depending upon the type and location of antigen, DCs can activate two arms of adaptive immunity; 1) humoral activation from extracellular pathogens and toxins, and 2) cell-mediated immunity from intracellular pathogens or tumors (**Figure 1**). Humoral immunity is initiated by T-cell-dependent and -independent antigens, and leads to B cell activation, clonal expansion and antibody secretion (**Figure 1**). Cell-mediated responses, on the other hand, involve activation of cytotoxic T cells, NK, NKT and macrophages, for the purpose of destroying abnormal or infected cells.

DCs do not present antigens directly to T-helper cells of both arms but first internalize and process them as antigenic peptides that are presented by major histocompatibility complexes (MHCs) located on their surface. DCs can present antigen on both MHC class I and class II complexes depending upon the source (128). Antigens arising from an intracellular source and presented on MHC class I will activate CD8 (cytotoxic) T cells (facilitated by IL-12 and type 1 IFN); while antigenic peptides from an extracellular (or foreign) source are presented on MHC class II molecules and activate CD4 (helper) T cells (128). In contrast to MHC class II molecules, MHC class I are more ubiquitous and found on all nucleated cells, presumably, conferring an evolutionary advantage for wider interorgan communications and defense (53, 58, 142, 153). Importantly, DCs are generally classified as the master regulators of adaptive immunity because they have greater capacity to transfer the three signals (see above) to initiate the response compared to any other APC (128). It is also noteworthy, that mitochondrial metabolism is key for the regulation of the effector response. In pro-inflammatory cells, such as M1 macrophages and activated T and B cells, the metabolic energy in the form of ATP is generated largely by glycolysis, while in regulatory cells, such as M2 macrophages or regulatory T cells, energy is generated by increased reliance on mitochondrial function and beta-oxidation (131).

### 5) Resolution Strategies to Halt the Inflammatory Process and Promote Healing

Studies in recent years have unequivocally shown that resolution of inflammation is an actively controlled processes rather than a passive procedure in which the proinflammatory immune cascade in inflammation simply fizzles.

Markus F. Neurath (2019) (154) p627

After the threat has been neutralized, immune cells begin to switch effector status and metabolism to their healing phenotypes or they undergo programmed apoptosis. Macrophages switch to an anti-inflammatory M2 phenotype (155–159), neutrophils undergo self-destruct (160), and T cells change their population from killer Th1 to a Th2 healing phenotype (via IL-10 and IL-4 produced by DCs) (141, 161). Th2 cytokines also help to maintain the presence of the M2 phenotype (155–159). In addition, DCs induce the production of T regulatory cells that suppress the destructive pro-inflammatory activities of Th1 cells by secreting cytokines, such as TGF-β1, IL-10 and IL-35, and enhance M2 responses to promote cell clearance (47, 160, 162, 163). Collectively, these changes limit inflammation and create a permissive healing environment (**Figure 3**).

As part of the healing processes, a further distinction is often made between anti-inflammatory and pro-resolving responses (126, 164–166). The term “pro-resolving” generally refers to the active suppression of the inflammatory *processes*, such as recruitment of immune cells, apoptosis and clearance of cell debris, whereas the anti-inflammatory targets refer more to specific inhibitory or blocking actions of *one or more pathways*, which may involve inhibiting PRRs, signal transduction, transcriptional/translational shuttling, and/or gene expression (**Figure 3**) (77, 165, 167). Molecules that fulfill the criteria of pro-resolving mediators include specialized lipid mediators (lipoxins, resolvins, protectins, and maresins) (126, 166), proteins and peptides (annexin A1, adrenocorticotropic hormone), gaseous mediators (H_2_S and CO), purines (adenosine), as well as neuromodulators (acetylcholine and other neuropeptides) released under the control of the vagus nerve (126, 165, 167–169).

Inflammatory inhibitors, on the other hand, include inhibitors or antagonists of TLR, NF-κB, TNF-α, Type I interferons (IFN-α and β), Type II IFN-γ, IL-1β, TGF-β1, and suppressor of cytokine signaling (SOCS) proteins, which have attracted much interest because of their ability to inhibit cytokine signaling pathways, and many others (111, 170). Notwithstanding the separation of *process* and *pathway*, much overlap exists between anti-inflammatory mediators and pro-resolving mechanisms. The successful development of new anti-inflammatory drugs with pro-resolving properties will likely involve inhibition of a “systems” process rather than targeting a single pro-inflammatory cytokine or pathway (see below).

## CNS Control of Inflammation: Crosstalk Between the Nervous and the Immune System

The defense of the organism against deleterious agencies, which is at first confined to the phagocytic mechanisms and the somatic system of nerves, by and by spreads to and is undertaken by the psychical nervous apparatus … One function of these psychical cells has been to develop a complete science for the defense of the organism against hostile influences.

E. Metchnikoff (1893) (18) p195

A topic that is often overlooked in discussions of immune response is the regulatory role of the CNS. Over a hundred years ago Metchnikoff recognized the importance of the “psychical nervous apparatus” in the host’s defense (above quote) (18). Today, the evidence suggests that the CNS interacts with immune cells in a bidirectional manner through shared receptors and neurotransmitters (126, 171–173). Macrophages, neutrophils, mast cells, DCs, blood monocytes, B cells and T lymphocytes all express many neurogenic receptors, including dopamine, adrenergic (β -2, α-1 and 2), glutamate acetylcholine and serotonin receptors (146, 148, 171, 173, 174). At the site of infection or injury, this would allow short-range communication between immune cells and recruitment of local neuronal signals to fine tune the immune response (172, 173). Dopamine (DA) bidirectional pathways are particularly interesting because dopamine has emerged as a fundamental regulator of inflammation (175). To this end, the primary and secondary lymphoid organs are highly innervated by sympathetic nerve cell terminals that store DA (176). Uncontrolled activation of dopamine pathways is believed to contribute to excessive inflammation and longer-term autoimmune pathologies (176). Recent studies have shown that the CNS-immune crosstalk is linked to increases in circulating neurohormones and cytokines, such as IL-1β and IL-6, which in turn are known to activate the HPA axis (173).

We have argued elsewhere that acute changes in the HPA axis-sympathetic-parasympathetic outflows are associated with immune dysfunction following different trauma states and sepsis (11, 12), and responsible for “low-level” persistent inflammation in most chronic inflammatory diseases, such as osteoarthritis and cardiovascular diseases (61, 64). Recent studies further demonstrate that the vagus nerve can modulate the host immune response after an infectious or sterile barrier breach (126, 168, 173), which may also have feedback inputs from changes to the CNS-gut-microbiome-immune axis (61, 64, 177). Increased vagal outflow to the spleen leads to reduced activation of circulating neutrophils by modulating the expression of CD11b (146). This indicates that the CNS can control neutrophil recruitment, *and may be vitally important for timely resolution of inflammation after a barrier breach*. Some catecholaminergic/cholinergic neurotransmitters can also modulate centrally-driven T cell-mediated immunity, although the underlying mechanisms are not well understood. To sum up, peripheral nerve cell interactions with immune cells is emerging as a major player in controlling the type and extent of inflammation and the immune response (146, 173). From a clinical perspective, modulating the different neuro-immune circuits, and their interactions, can potentially modify inflammatory processes and pathways, that may lead to better outcomes after major trauma, infection or disease.

## Developing New Therapies to Reduce Excessive Inflammation: Choosing the Right Animal and the Conditions for Safety and Translation to Humans

Achieving FDA approval for only one-in-ten drug indications that enter the clinic is a concerning statistic for drug developers, regulators, investors and patients.

Hay and colleagues (2014) (178)

Currently, there are few safe and effective drug therapies targeting excessive inflammation and immune dysfunction after trauma or infection. The widely used non-steroidal anti-inflammatory drugs (NSAIDs), COX-2 inhibitors and TNF-α inhibitors do not appear to be pro-resolving, and may in fact exacerbate the resolution process (166). The reasons for lack of progress in drug development in this area is complex. The 90% failure rate of new drugs reaching FDA approval is one of the major impediments because of the high risks of failure and high costs (178). Furthermore, if a drug does reach approval, around 30% have been shown to have a postmarket safety event (139, 179). This adverse event statistic is startling given the high level of scrutiny from independent institutional review boards and pre-market safety and regulatory oversight guidelines imposed by the FDA. We propose at least five reasons for “translational failure”:

1. Choice of animal model and gut microbiome status
2. Male animal species and human study bias
3. Heterogeneity of the human response to infection or injury
4. Poor clinical trial design in a real-world environment
5. Ignoring the systems approach

In our view, choosing the right animal model is one of the single determinants for failure of new drugs to translate (61, 62, 177, 180, 181). An overlooked variable is the composition of gut microbiome. In the 1960s, disease was an unwanted variable in small animal experiments, and potentially harmful bacteria were selectively bred out of animal colonies (61, 62, 177). These animals are called specific pathogen-free (SPF) and the gut composition varies from institution to institution, and different animal suppliers. The problem with modifying gut microbiomes is that the microflora ‘mix’ can lead to host immune alterations and responses that are often not representative of the human condition (61). In a landmark study, Beura and colleagues demonstrated that SPF adult mice have “immature” immune systems that were more prone to inflammation and infection than conventionally-bred mice (182). Their SPF mice lacked effector-differentiated and mucosally-distributed memory T cells, and when co-housed and bred with pet store mice, their immune system shifted closer to adult humans (182). Similarly, we have reported that SPF animals are not suitable for studying traumatic injury or hemorrhagic shock (62, 177).

Male bias in animal and human studies is another important variable affecting drug translation. In the past, the exclusive use of male animals assumes that male and female animals are biologically identical (183, 184). This assumption is false (137, 183, 185). Women have twofold higher mortality after equivalent burn injury than men, and have lower incidence of sepsis and mortality after major surgery or blunt force trauma (186). Women also have different responses to many FDA-approved drugs than men, including anti-inflammatory drugs, and in some cases with adverse outcomes (184, 187–189). Problems with translation also depend on the heterogeneity of the population including socio-economic, age and health status, that can impact clinical trials (179, 181, 190). While randomized control trials are considered the “gold standard”, they may not always mimic real-life treatment situations because of their strict inclusion/exclusion criteria, and highly controlled settings (191). In addition, many trials suffer from poor design and low statistical power to support primary and secondary endpoints (184, 191).

Another major contributor to “translational failure”, that receives little attention, is the flawed practice of drugs targeting single nodal steps (61, 62, 180). Targeting individual pro-inflammatory cytokines, for example, or any single step along a signaling pathway, ignores the importance of the system. Single-nodal thinking rarely solves a medical problem unless the site is believed to be a central hub or intersection point. The IL-1 receptor has been proposed to be such a site, and while anakinra (IL-1 antagonist) has an excellent safety record, further trials are required to demonstrate its clinical efficacy after an infection and trauma (192, 193). Reductionism in scientific discovery is important in breaking a system into its constituent parts, and studying them, however, *it does not do away with the system* (61, 62, 177). This flawed way of thinking, we believe, is responsible for a high failure rate of translating promising new drugs, and why there are so many failed clinical trials (194). A systems approach is much more likely to increase animal to human success of translation.

## Toward a System-Based Drug to Protect Against Excessive Inflammation After Infection And Sterile Injury

What we anticipate seldom occurs; what we least expect generally happens.

Benjamin Disraeli (1804–81), Henrietta Temple (3)

### Brief History: Teaching Old Drugs New Tricks

Twenty years ago, the first author (GPD) asked if it was possible to pharmacologically manipulate the human heart to operate more like the heart of a natural hibernator for improved protection during adult and pediatric cardiopulmonary bypass or valvular surgery (2, 3, 195). Within 10 years, high dose ALM cardioplegia was translated from isolated rat heart experiments into human cardiac surgery. We chose adenosine (A) to inhibit the sinoatrial node and reduce the atrial and ventricular action potential (AP) duration (A1 receptor subtype and A1 linked opening of K_ATP_ channels), lidocaine (L) to reduce AP amplitude by arresting Na^+^ fast channels, and magnesium (M) to stabilize the membrane and protect against reperfusion arrhythmias (196). We theorized this strategy would arrest the heart at its resting membrane potential and avoid the use of commonly used high potassium, which depolarizes the membrane and promotes ‘ischemic’ injury currents (197, 198). Two prospective, randomized, clinical trials have shown the ALM cardioplegia to be superior to high potassium cardioplegia with less days in hospital (199, 200). After surgery, the heart is reanimated in sinus rhythm with 10-times lower concentrations of ALM in a heart. This resuscitation strategy led to a second idea; namely, the application of ALM to resuscitate and protect the heart after major trauma such as hemorrhagic shock, cardiac arrest, heart attack and stroke and trauma of surgery itself (3).

### Adenosine, Lidocaine, and Mg^2+^ Immune-Modulatory Functions

We will now briefly review our efforts to develop an intravenous (IV) fluid therapy comprising adenosine, lidocaine and Mg^2+^ (ALM) *that uniquely protects against trauma and infection, and may be useful as an immune-modulatory agent for coronavirus 2 (SARS-CoV-2), and other viral challenges.* Our early experiments focused on traumatic injury (139, 201–205) and subsequently we showed the same therapy protects against sepsis and endotoxemia in different animal models (3, 206, 207). The individual effects of A, L or M also have been shown to confer some protection, but not to the same extent as ALM combined (12, 208). Some of these effects on immune cells, and key regulatory sites, are listed in **Tables 2** and **3**. Each drug exerts anti-inflammatory and immunoregulatory effects from molecular to cellular and tissue-level responses (209–214), including 1) differential PRRs expression (e.g. TLRs) and activity of cell surface receptors, 2) the activation of signal transduction pathways and 3) the transcription factors responsible for regulating inflammatory cytokine production, and preservation of cellular energy metabolism (3, 211, 215–238) (**Tables 2** and **3**). In addition, A, L or M individually have been shown to support endothelial-mediated inflammatory/immune functions (3, 239), however, they do not do so to the same extent as ALM combined (12, 208). The precise mechanisms of how ALM confers a survival benefit at the level of the immune system, and the intersection between trauma and infection, are not known (3, 139). What follows is a characterization of the ALM survival phenotype and why it may be applicable to reduce inflammation from a viral attack.

**Table 2:** Summary of the effects of adenosine, lidocaine and magnesium alone on immune cells.

Immune Cell Type	Adenosine	Lidocaine	Magnesium
Macrophage	Inhibits M1 (induced by IFN-γ plus TNF-α or TLRs). Enhances M2 (anti-inflammatory).	Inhibits M1 (↓ NO, CAT-2 & IL-1β from LPS-activated macrophages).	High concentrations ↓ M1- type inflammation, enhance M2.
Dendritic Cell	Extracellular ATP activates. Downregulates & dampens T cell activation & cytokine secretion.	Suppresses activation of DCs via ↓ cell-mediated Th1 cell differentiation (↓IL–6, TNF-α and IL–12) and ↑ IL-10 in LPS-activated DCs).	High concentrations suppress Langerhans cell functions *in vitro.*
Neutrophils	Inhibits neutrophil adhesion & transmigration.	Inhibits by ↓ protein kinase. Inhibits superoxide release by ↓ integrin-mediated outside-in signaling & GPCR function (211).	Reduces respiratory burst (212).
T Cells, CD8^+^ (cytotoxic cells)	Inhibits effector differentiation (209).	↓ T cell proliferation and cytokine secretion partly by inhibition of NF-κB signalling (211). ↑ inhibitory effect on the T cell-mediated immune response during major surgery.	Regulates cytotoxic functions (213). Decreased levels activate T cells (214).
T Cells, CD4^+ ^ (helper cells)	A2A inhibits IFN-γ to limit T cell activation & secondary M1 activation in inflamed tissues.	Abrogates T cell proliferation & suppresses expression of the T cell-derived proinflammatory cytokines IL-2, TNF-α & interferon (IFN)-γ via NF-κB-mediated inhibition of mRNA expression (211).	
NK cells	Inhibits proliferation, maturation, & cytotoxic function (210).	Inhibitory at high concentrations. Low concentrations ↑ killing activity.	Regulates cytotoxic functions. Decreased levels activate NK cells (214).

**Table 3:** Effects of Adenosine, Lidocaine and Magnesium alone on Toll-like receptors, NF-κβ, TNF-α and inflammasome activity.

	Adenosine	Lidocaine	Magnesium
General Properties, Receptors & Functions	Adenosine exerts its actions via four distinct G protein-coupled receptors (A1, A2A, A2B and A3), & different downstream signaling pathways (3). The A1 and A3 receptors are coupled to the G_i_ family of proteins resulting in decreased cAMP. A2A receptors are high affinity Gα_s_-coupled receptors that increase intracellular cAMP. Adenosine has potent anti-inflammatory, vasodilator, anti-ischemic, & cardio- and neuro-protective properties, & is involved in wound healing. Adenosine receptors are widely expressed in the CNS, heart, liver, spleen, muscle, kidney, lung & immune cells (3).	Lidocaine acts via blocking Na^+^ fast channels & non-Na channels, including specific receptors within G-protein- coupled receptor family (primarily via the Gaq subunit) (226). Lidocaine is an analgesic, anti-arrhythmic & has anti-inflammatory properties on various cell types, including monocytes, macrophages & neutrophils. It also has anti-ischemic properties in many tissues (3).	Mg^2+^ exerts its effect on voltage-dependent binding sites & receptor channels (e.g. NMDA receptor) and as a cofactor for energy-linked enzymatic reactions. Mg^2+^ is a natural calcium antagonist. & a potent calcium channel inhibitor, affecting Ca^2+^ regulation in immune & non-immune cells (233).
Toll receptors (TLRs)	Reduces cell surface expression of TLR4 & TNF-α in human macrophages in response to LPS or hyaluronic acid stimulation (215, 216).	Downregulated TLR4 & NF-κβ & inhibited induction of IL-1β, IL-6, IFN-γ, TNFα in liver & kidney in rat model of LPS-induced sepsis. NF-κB also downregulated in the heart, with increased survival (227). Similar results have been reported after sterile injury (228).	No studies showing a direct or indirect effect of Mg^2+^ on TLRs.
NF-κβ	Potent inhibitor of the NF-κB pathway in B cell antigen receptor signaling, & downregulates TLR4 in splenic B lymphocytes (217). The suppression appears to target blocking the phosphorylation & subsequent degradation of the inhibitor of NF-κβ (218). Blocks activation of NF-κB & enhances AP1 binding activity in ischemic rat heart (219). Downregulates TLR4/NF-κB expression & prevents myocardial ischemia-reperfusion injury (220).	Inhibits T cell proliferation & suppresses expression of the T cell-derived proinflammatory cytokines IL-2, TNF-α & IFN-γ via NF-κB-mediated inhibition of mRNA expression (211). Attenuates LPS-induced acute lung injury by inhibiting NF-κB activation (229). Reduces progression of cerebral ischemia-reperfusion injury in rats by suppressing the activation of NF-κB, p65 & p38 MAPK (230).	Increased basal IĸBa levels in isolated blood mononuclear cells, & upon TLR stimulation was associated with reduced NF-κB activation and nuclear localization (234). Inhibits LPS-induced inflammatory molecules production & NF-κB activation in activated RAW264.7 cells. May involve antagonizing calcium by inhibiting the L-type calcium channels, or both (235). Similar effects reported in activated microglia (236).
TNF-α	Inhibits TNF-α by signaling via A2A & A2B receptors in macrophages (221). Inhibits TNF*-*α in adult rat ventricular myocytes, & in human heart following ischemia-reperfusion injury (222–224).	Prevents TNF-α-induced secretion of the proinflammatory mediators IL-1β & IL-8 from intestinal epithelial cell lines, via down-regulation of NF-κB translocation to the nucleus (211). Decreased TNF-α expression in lung & systemically in pigs undergoing lung resection surgery (231).	Inhibits expression of TNF-α via PI3-K/AKT & NF-κB-dependent mechanisms (237).
Inflammasome	Key regulator of inflammasome activity via the A2A receptor (225).	Suppresses the generation of IL-1β in macrophages, & may block inflammasome-NF-κB-caspase-1 activation (232).	Inhibits NLRP3 inflammasome, IL-1β upregulation, and pyroptosis, & believed linked to decreased intracellular calcium levels (238).

### Adenosine, Lidocaine, and Mg^2+^ Defends Against Infection and Trauma

A 4-hour infusion of ALM after polymicrobial sepsis led to 6-day survival (without antibiotics), while controls died from different levels of immune dysfunction on days 2 and 4 (3, 207). ALM survival was associated with suppression of the systemic inflammatory response. Interestingly, blood IL-1β levels remained at baseline levels, which we argued was associated with inhibition of the inflammasome (207). As discussed, IL-1β is a potent inducer of inflammation *via* the inflammasome and activation of NF-κB (112, 240). In another porcine model of LPS endotoxemia, ALM infusion blunted inflammation and bolstered the cardiovascular system to produce a high flow, hypotensive, vasodilatory state with improved O_2_ delivery over a 4-hour period (241).

With respect to trauma, the ALM therapy suppressed inflammation after hemorrhagic shock, major surgery or traumatic brain injury (139, 201–205, 242). After hemorrhagic shock, ALM extended survival to 3 days, which was associated with improved cardiac function, correction of coagulopathy, preservation of platelets, and differential expression of the master genes of metabolism (ampk, sirt-1, PGC-1α and mtCO3). Metabolic genes were upregulated in heart and brain and downregulated in the periphery (liver, gut) at 3 days. Increased expression of sirt-1 in heart is clinically relevant because sirt-1 activation has been shown to inhibit NF-κB signaling, and reduce inflammation (243). Similarly, upregulation of heart mtCO3 indicates improved mitochondrial metabolism by improving cytochrome c oxidase to drive ATP synthesis (204). TFAM (transcription factor A, mitochondrial), a gene involved in mitochondrial biogenesis, was also significantly increased in heart and brain at 3 days. From a systems perspective, the ALM survival phenotype appears to be associated with changes to the CNS, with downregulation of sympathetic activation and upregulation of parasympathetic outflow during recovery (3). As discussed above, an increased reliance of parasympathetic outflows is consistent an anti-inflammatory survival phenotype (146).

### Future Studies and Outstanding Questions

Currently, we don’t know how and when the host “switches” from an injury phenotype to a survival one, or if protection can be extended from 3 to 7 days, or longer. Neither do we know if the therapy confers the same whole-body protection if administered at later times *after* insult? Other questions include what are the underlying mechanisms responsible for ALM’s ability to reduces the “cytokine storm”? What roles do different immune cells, such as macrophages and neutrophils, and platelets play in inflammatory suppression? What is the role of adenosine receptors, lidocaine Na^+^ fast channels and magnesium ions to PRR signal activation and transduction of inflammatory pathways? What role does ALM play in regulating intracellular Ca^2+^ and the metabolic pathways involved in maturation of the immune response? Are there common intersection points at NF-κB in the regulation of the immune response? How is ALM’s dual protection capability linked to altering CNS-cardiac-endothelial-mitochondrial coupling and improved survival after trauma and infection?

### Adenosine, Lidocaine, and Mg^2+^ as a System-Based Therapy

**Figure 5:**
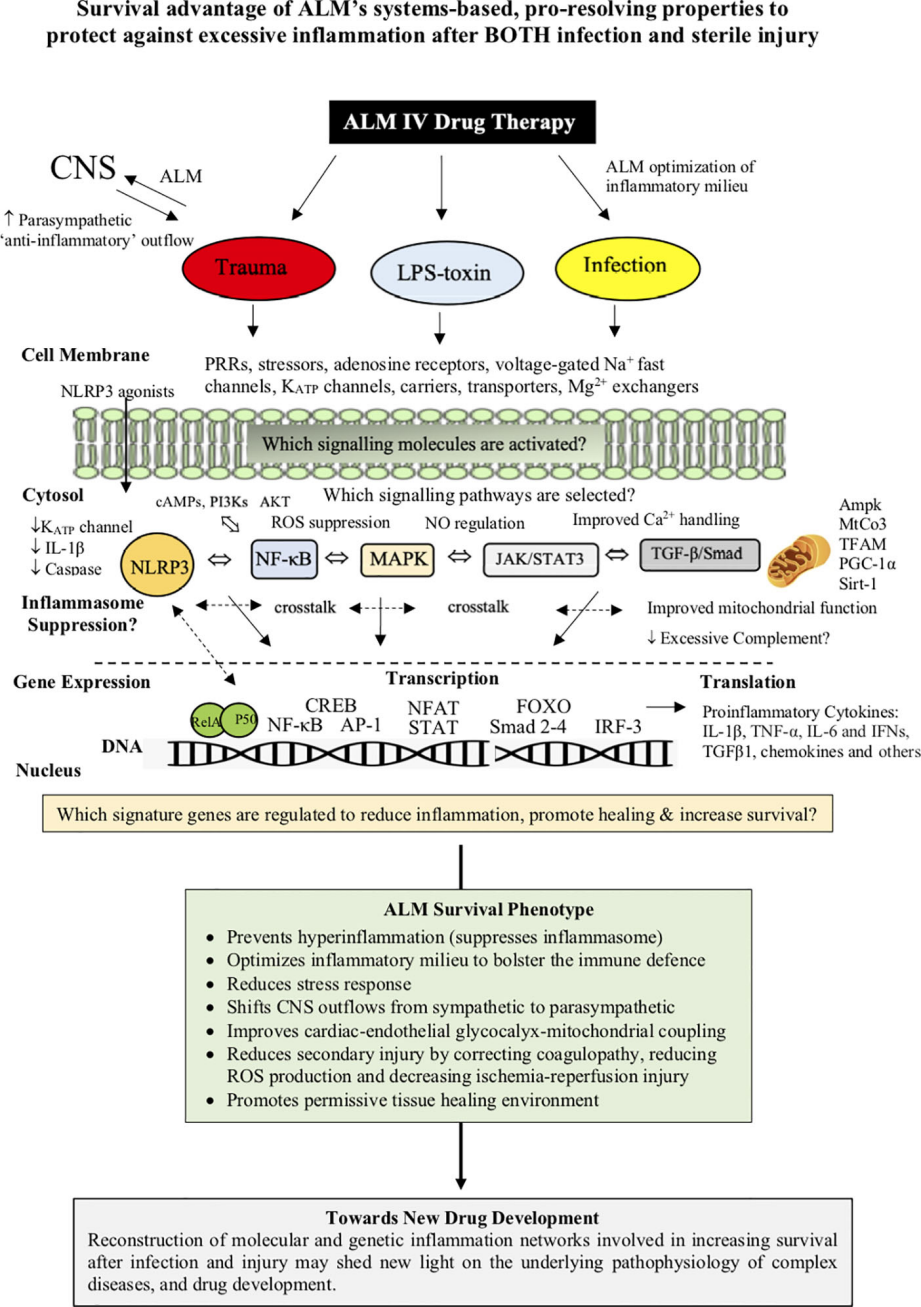
ALM therapy drug development has revealed a duality of protection against infection/toxin and major trauma. The ALM survival phenotype involves an early genetic switch and reprograming of the whole body to provide CNS-cardiac-endothelial and mitochondrial support and early bolstering of the immune system with suppression of hyperinflammation and secondary injury processes. The underlying mechanisms have been shown to involve the differential expression of the master genes of metabolism, however, it is not known what transcriptional signaling mechanisms are involved in ALM suppressing of inflammation after infection or trauma. Genomic and proteomic analysis arrays may help unmask the changes in patterns of gene expression, and the nature of the ALM survival switch that protects against an infection/toxin or major trauma.

On the basis of the available evidence, ALM appears to confer a “systems’ effect” of dual protection, which has led us to propose a Systems Hypothesis of Trauma (SHOT) (11, 12). SHOT is discussed in detail elsewhere and begins with improved CNS control of cardiovascular function, support of endothelial-glycocalyx integrity, and improved O_2_ delivery and mitochondrial metabolism, that is not replicated with the individual actives A, L or M (3, 11, 12). ALM’s dual protection capability may offer a unique opportunity to control excessive inflammation and secondary injury in patients suffering infectious diseases, such as COVID-19 presenting with cardiac, metabolic, pulmonary and immune dysfunction (193, 244, 245) (**Figure 5**).

## Conclusions

For much of the 20^th^ century, the different models of immunity rarely included Metchnikoff’s theory of inflammation and phagocytotic defense. Today, inflammation is viewed as a key component of innate and adaptive immunity that creates a milieu that removes and resolves infectious and non-infectious threats. The type and extent of immune response is determined by the mix of cytokines and other neural and inflammatory mediators that determine the selection, activation, recruitment and fate of immune effector cells. Although inflammation is essential for threat neutralization and healing, if left unresolved, it can lead to immune dysfunction and further tissue damage with coagulopathy, endothelial dysfunction, mitochondrial dysfunction, organ failure and death. We have been developing an intravenous (IV) fluid therapy comprising adenosine, lidocaine and Mg^2+^ (ALM) that confers a survival advantage by preventing excessive inflammation associated with sepsis, endotoxemia and sterile trauma. ALM may provide a therapeutic option for treating COVID‐19.

## Author Contributions

All authors contributed to the article and approved the submitted version.

## Funding

This work was supported by USSOCOM, USAMRMC proposal SO150053 under Award No. W81XWH-USSOCOM-BAA-15-1. The opinions, interpretations, and conclusions are those of the authors and are not necessarily endorsed by the US Department of Defense.

## Conflict of Interest

GPD is the sole inventor of the ALM concept for cardioplegia and trauma.

The remaining authors declare that the research was conducted in the absence of any commercial or financial relationships that could be construed as a potential conflict of interest.
